# Increasing Circulation

**Published:** 1916-09

**Authors:** 


### Increasing Circulation.

The Texas Medical Journal has determined to grow, and has begun the necessary treatment under the advice of experts in circulation. It has determined that there shall be no dwarfed, stunted existence for it; that it will stand erect and in its stature will look other medical publications squarely in the eyes. Some of them will have to look up to it in the future unless they also try some radical treatment. The prescription used by The Texas Medical Journal is not a secret; it is not proprietary; it is not patented; and it is strictly ethical. Told in a few words, The Texas Medical Journal is furnishing the medical profession with the ablest articles that can be obtained on live professional subjects, and is leaving nothing undone to interest the strongest men in contributing such articles. It does not wait until the profession is no longer interested in the themes discussed, but when a disease or diagnosis is attracting attention this journal at once gets busy to find who knows most about it, and induces those who do know, or who think they know, to tell others. The secret of all success in any business or profession is in just being alive; do not misunderstand, it is not in being just alive, but in just being alive. Too many physicians are just alive, and they are, of course, failures. Too many are not alive enough to take an up-to-the-day medical journal, and even if they could be induced to take it they could not be persuaded to read and study it.

But there are enough live men in the profession in the South to appreciate what is being done to make The Texas Medical Journal a success, and they are taking it by the hundreds. It is some feat to add a thousand new subscribers to a medical journal in the dull months of summer, but that is just what has been done, and the plans are already made for another thousand this month. If you appreciate what is being done to this end, speak a good word for the Journal and its editor and help in the good work. You will not only favor the publication, but will be doing your fellow physicians a good service.

---

Many a man thinks he is old, and played out, and tired of the world and all it contains, when he is merely lazy or lonely.—*Medical Summary.*

